# A model for the conformational activation of the structurally quiescent metalloprotease ADAMTS13 by von willebrand factor

**DOI:** 10.1074/jbc.M117.776732

**Published:** 2017-02-16

**Authors:** Kieron South, Marta O. Freitas, David A. Lane

**Affiliations:** From the Centre for Haematology, Imperial College London, London W12 ONN, United Kingdom

**Keywords:** ADAMTS13, allosteric regulation, hemostasis, protein conformation, von Willebrand factor

## Abstract

Blood loss is prevented by the multidomain glycoprotein von Willebrand factor (VWF), which binds exposed collagen at damaged vessels and captures platelets. VWF is regulated by the metalloprotease ADAMTS13, which in turn is conformationally activated by VWF. To delineate the structural requirements for VWF-mediated conformational activation of ADAMTS13, we performed binding and functional studies with a panel of truncated ADAMTS13 variants. We demonstrate that both the isolated CUB1 and CUB2 domains in ADAMTS13 bind to the spacer domain exosite of a truncated ADAMTS13 variant, MDTCS (*K_D_* of 135 ± 1 0.1 nm and 86.9 ± 9.0 nm, respectively). However, only the CUB1 domain inhibited proteolytic activity of MDTCS. Moreover, ADAMTS13ΔCUB2, unlike ADAMTS13ΔCUB1-2, exhibited activity similar to wild-type ADAMTS13 and could be activated by VWF D4-CK. The CUB2 domain is, therefore, not essential for maintaining the inactive conformation of ADAMTS13. Both CUB domains could bind to the VWF D4-CK domain fragment (*K_D_* of 53.7 ± 2.1 nm and 84.3 ± 2.0 nm, respectively). However, deletion of both CUB domains did not prevent VWF D4-CK binding, suggesting that competition for CUB-domain binding to the spacer domain is not the dominant mechanism behind the conformational activation. ADAMTS13ΔTSP8-CUB2 could no longer bind to VWF D4-CK, and deletion of TSP8 abrogated ADAMTS13 conformational activation. These findings support an ADAMTS13 activation model in which VWF D4-CK engages the TSP8-CUB2 domains, inducing the conformational change that disrupts the CUB1-spacer domain interaction and thereby activates ADAMTS13.

## Introduction

The large, multidomain glycoprotein Von Willebrand factor (VWF)^3^ recognizes exposed collagen at sites of vascular damage through its surface-exposed A3 domain (1–3). Once tethered to collagen, rheological shear forces induce a conformational change (4). The globular conformation, adopted under conditions of low shear, unfolds to reveal its binding site for the platelet GpIbα receptor within its A1 domain (5) and to enable platelet capture.

VWF, expressed in endothelial cells, multimerizes in the Golgi apparatus and is stored in Weibel-Palade bodies as heterogeneous high molecular weight multimers (as large as 20–40-mers) before being released into the plasma (6–9). Once in circulation, it is the highest molecular weight multimers that exhibit the most hemostatic potential. It is the presence of these “ultra large” VWF multimers, caused by ADAMTS13 deficiency, that initiates the formation VWF-platelet microthrombi (10), which characterize thrombotic thrombocytopenic purpura (TTP). Imbalance of the VWF/ADAMTS13 axis has also been suggested to play a role in both ischemic stroke and myocardial infarction (11–14).

The multimeric size and hemostatic potential of VWF is regulated through a multistep mechanism of proteolysis of its A2 domain by the metalloprotease ADAMTS13 (15, 16). This process begins with a positioning interaction between the C-terminal domains of ADAMTS13 and the C-terminal D4-CK domains of globular VWF (17, 18). VWF is maintained in its globular conformation by structural elements of the A2 domain (19–23), with the ADAMTS13 cleavage site inaccessible. Upon shear-induced unfolding of the VWF A2 domain, there is a progressive exposure of distinct binding exosites that are engaged by complementary exosites in the ADAMTS13 spacer, cysteine-rich, disintegrin-like, and metalloprotease domains (24–29). The newly exposed scissile bond of VWF (Tyr-1605–Met-1606) is positioned for cleavage by the docking of its P1′, P1, and P3 residues into complementary subsites in the protease domain (30).

The importance of ADAMTS13 conformation in regulating VWF proteolysis has been recently investigated. The proteolytic activity of ADAMTS13 against VWF73 is enhanced in the truncated variant MDTCS (31, 32), suggesting an autoinhibitory role of the C-terminal domains of ADAMTS13. A globular conformation of ADAMTS1=3, maintained by binding between its spacer and CUB domains (31), is suggested to be facilitated by up to three flexible linker regions within the C-terminal tail (33). In a gain of function (GoF) ADAMTS13 variant (R568K/F592Y/R660K/Y661F/Y665F), which exhibits enhanced proteolytic activity (34), this autoinhibitory interaction is disrupted, and an open conformation is adopted (31). The VWF D4-CK domain region is essential for enhancing activity of ADAMTS13 (31, 32), indicating that the positioning interaction between VWF D4-CK and ADAMTS13 (17) actuates the conformational change required to expose the functionally important spacer domain exosite. Conformational quiescence of ADAMTS13 hides the exosites predominantly recognized by TTP autoantibodies (31) and may also serve to prevent off-target proteolysis of plasma proteins, as it has now been demonstrated that conformationally active ADAMTS13 is capable of proteolysing fibrinogen (35).

To further explore the molecular basis of conformational activation of ADAMTS13, we have prepared and studied a panel of ADAMTS13 domain deletion variants. We have employed binding and functional assays to determine the role of the ADAMTS13 C-terminal domains in its activation by VWF D4-CK. We propose that CUB1 binding to the spacer domain forms the autoinhibitory basis of its quiescent conformation. Moreover, several distinct interactions between VWF D4-CK and ADAMTS13 have been identified, with the TSP8 domain of ADAMTS13 proving essential for VWF D4-CK-induced activation.

## Results

### CUB1 and CUB2 exhibit independent binding to the spacer domain

We first conducted a preliminary co-immunoprecipitation investigation of the binding between the isolated CUB1 and CUB2 domain fragments and the truncated ADAMTS13 variant, MDTCS. Both CUB1 and CUB2 were shown to interact with WT MDTCS in solution with an affinity that was sufficient to allow specific co-immunoprecipitation of the CUB fragments when MDTCS was pulled down. As expected, binding of neither CUB fragment to the GoF MDTCS variant was observed.

**Figure 1.**
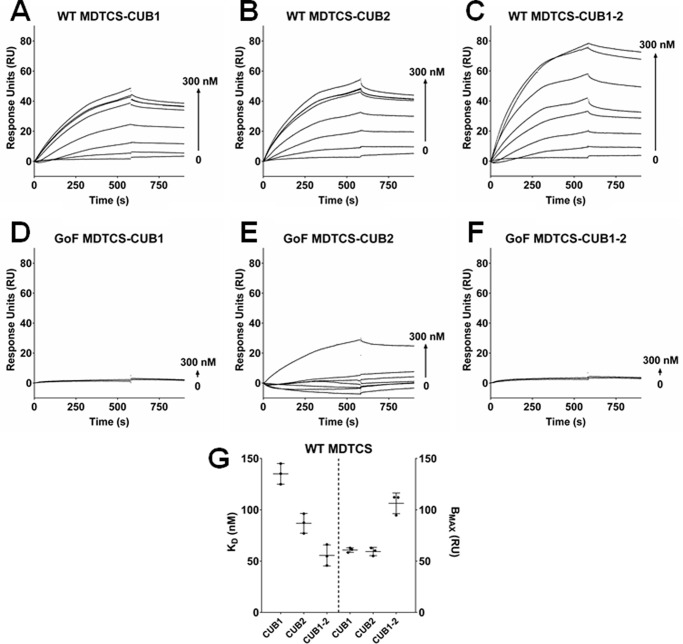
**Concurrent binding of CUB1 and CUB2 had an additive effect on their affinity for the spacer domain of ADAMTS13.** Binding between WT MDTCS (*A–C*) or GoF MDTCS (*D–F*) to the immobilized C-terminal domain fragments CUB1, CUB2, and CUB1-2 was determined by SPR. Results are representative of three independent experiments. *G*, global analysis was used to determine *K_D_*, and B_max_ values were used for the binding between WT MDTCS and the CUB domain fragments (mean ± S.D., *n* = 3).

These findings were corroborated by surface plasmon resonance analysis in which MDTCS variants (WT or GoF) were immobilized on a sensor chip (Fig. 1). Both CUB1 and CUB2 bound to WT MDTCS with moderate affinities (Fig. 1, *A* and *B*), *K_D_* values of 135 ± 10.1 nm and 86.9 ± 9.0 nm, respectively (Fig. 1*G*). As expected, a higher affinity interaction was observed between WT MDTCS and the CUB1-2 fragment (Fig. 1*C*), with a *K_D_* value of 55.6 ± 10.9 nm, similar to that reported previously (31). There was also an ∼2-fold increase in the B_max_ of binding between WT MDTCS and CUB1-2 compared with the isolated CUB1 and CUB2 domain fragments (Fig. 1*G*). In support of co-immunoprecipitation (co-IP) experiments, no binding between CUB1 or CUB1-2 and GoF MDTCS (Fig. 1, *D* and *F*) and minimal binding between high concentrations of CUB2 and GoF MDTCS were observed (Fig. 1*E*).

### CUB1-spacer binding maintains the autoinhibited ADAMTS13 conformation

**Figure 2.**
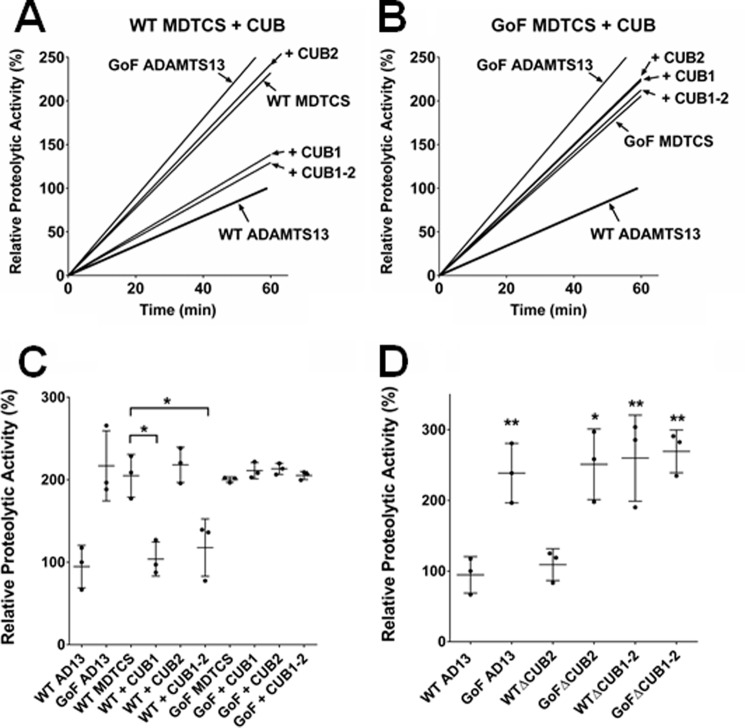
**CUB1 binding to the spacer domain maintained the closed conformation of ADAMTS13.** *A–C*, the FRETS-VWF73 assay was used to determine the inhibitory effect of the CUB domain fragments (CUB1, CUB2, and CUB1-2) on the relative proteolytic activity of the truncated ADAMTS13 variant MDTCS (WT or GoF). In each panel the activities of WT and GoF ADAMTS13 are included for reference. Normalized results (*A* and *B*) are representative of three independent experiments, and relative proteolytic activities (*C*) are presented as the mean ± S.D. (*n* = 3). *D*, the same assay was also used to determine the proteolytic activity of C-terminal-truncated ADAMTS13 variants (ΔCUB2 and ΔCUB1-2). Results are presented as the mean ± S.D. (*n* = 3). *, *p* < 0.05; **, *p* < 0.01.

As previously reported (31), WT and GoF MDTCS are both hyperactive, with activity ∼2-fold higher than that of WT ADAMTS13 and similar to GoF ADAMTS13 in FRETS-VWF73 assays (Fig. 2, *A* and *B*). The CUB1-2 domain fragment significantly inhibited the activity of WT MDTCS, but not the GoF variant, in this assay (Fig. 2*C*). Importantly, the isolated CUB1 fragment significantly inhibited WT MDTCS (*p* < 0.05) to a similar extent as CUB1-2, but the isolated CUB2 fragment was without effect, even when added up to a concentration of 10 nm (data not shown). This inhibitory influence of CUB1 was reflected in the activities of C-terminal-truncated ADAMTS13 variants. Truncation of WT ADAMTS13 after CUB1 (WTΔCUB2) did not significantly alter proteolytic activity in FRETS-VWF73 assays. However, removal of both CUB domains (WTΔCUB1-2) increased activity at a level similar to that of GoF ADAMTS13 (Fig. 2*D*). Truncation of GoF ADAMTS13 (GoF ΔCUB2 and GoFΔCUB1-2) did not alter its activity.

### Both CUB1 and CUB2 interact with the D4-CK domains of VWF but are not the major binding partners

**Figure 3.**
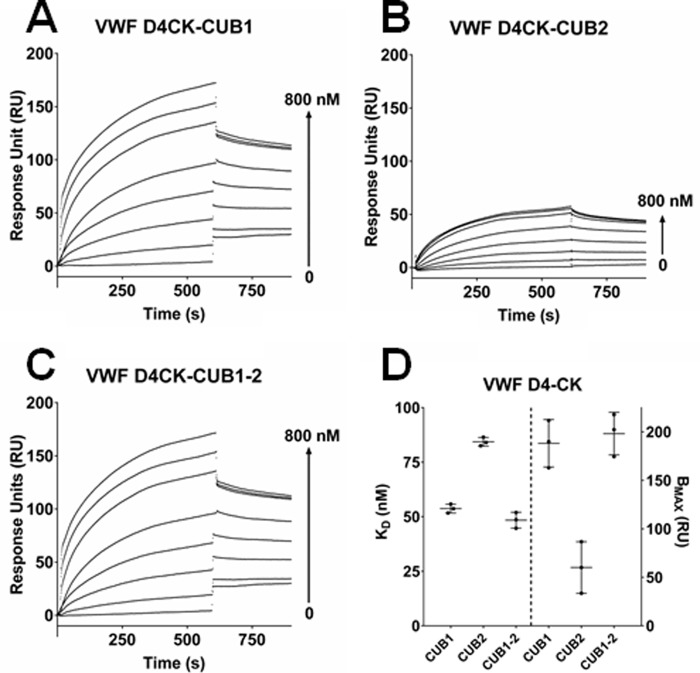
**VWF D4-CK interacted with CUB1 and CUB2.** *A–C*, binding between the VWF D4-CK domain fragment and the immobilized ADAMTS13 C-terminal domain fragments (CUB1, CUB2, and CUB1-2) was determined by SPR. Results are representative of three independent experiments. *D*, global analysis was used to determine *K_D_* and B_max_ values. Results are presented as the mean ± S.D. (*n* = 3).

SPR analysis has also revealed that both CUB1 and CUB2 (immobilized on a sensor chip) are able to bind the VWF D4-CK domain fragment (Fig. 3, *A* and *B*), with *K_D_* values of 53.7 ± 2.1 nm and 84.3 ± 2.0 nm, respectively (Fig. 3*D*). However, the capacity of binding between CUB2 and VWF D4-CK was markedly lower than that of CUB1, with B_max_ values of 60.1 ± 25.3 RU and 188 ± 24.5 RU, respectively (Fig. 3*D*). Furthermore, there was no increase in affinity between VWF D4-CK and the CUB1-2 domain fragment (Fig. 3*C*) compared with the isolated CUB1 fragment (*K_D_* values of 51.8 ± 1.9 nm and 53.7 ± 2.1 nm, respectively, *p* > 0.5).

**Figure 4.**
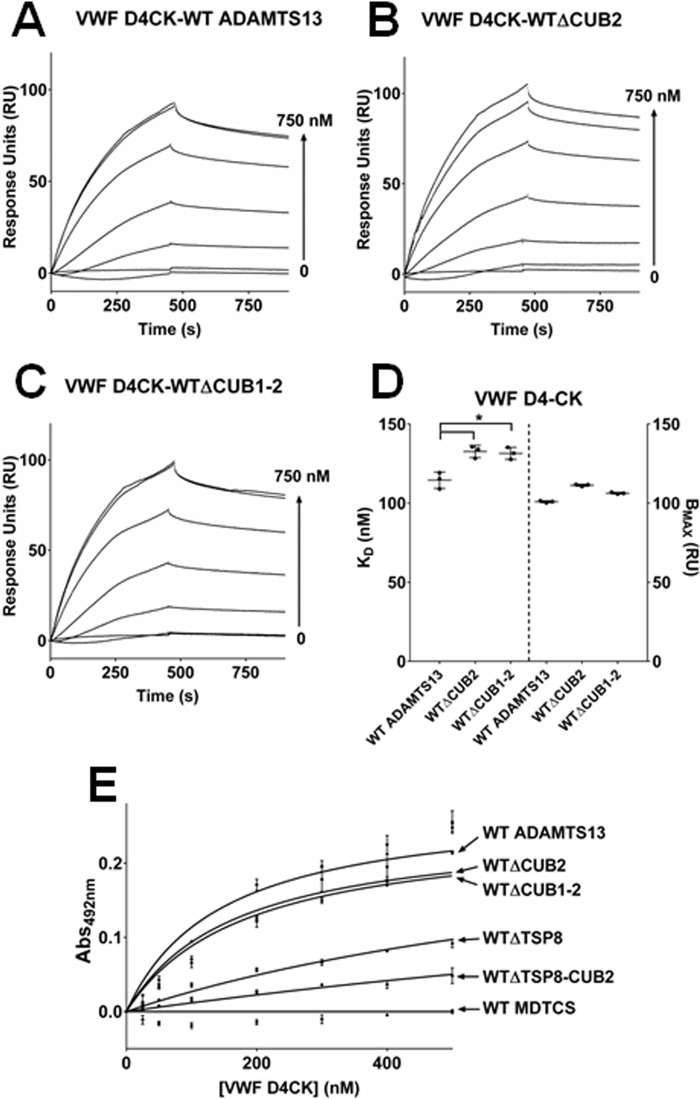
**The TSP8 domain but not the CUB domains was crucial for VWF D4-CK binding.** *A–D*, SPR analysis was also used to determine the binding between VWF D4-CK and immobilized ADAMTS13 truncation variants (WTΔCUB2 and WTΔCUB1-2). *, *p* < 0.05. *E*, the effect of sequential C-terminal truncation of ADAMTS13 on VWF D4-CK binding was further examined using an equilibrium plate binding assay. Results are presented as the mean ± S.D. (*n* = 3).

WT ADAMTS13, immobilized on the sensor chip, bound to the VWF D4-CK domain fragment (Fig. 4*A*) with a *K_D_* of 114 ± 5.2 nm (Fig. 4*D*), similar to that published previously (17). Surprisingly, truncation of ADAMTS13 after the CUB1 domain (WTΔCUB2) or after the TSP8 domain (WTΔCUB1-2) resulted in only a slight decrease in affinity for the VWF D4-CK domain fragment (Fig. 4, *B* and *C*) with the *K_D_* values (132 ± 3.9 nm and 131 ± 3.8 nm) but not the B_max_ values (111 ± 0.6 RU and 106 ± 0.5 RU) differing significantly (*p* < 0.05) from that of WT ADAMTS13 (Fig. 4*D*).

These results, suggesting that binding of ADAMTS13 to VWF D4-CK occurred in the absence of the CUB domains, were corroborated using an equilibrium plate binding assay (Fig. 4*E*). The *K_D_*_(APP)_ of binding between WT ADAMTS13 and VWF D4-CK, derived from these data (146 ± 15.5 nm), was somewhat higher than that determined by SPR. However, the same relative decrease in affinity for VWF D4-CK exhibited by the truncated variants WTΔCUB2 and WTΔCUB1-2 (*K_D_*_(APP)_ values of 167.0 ± 15.9 nm and 183.5 ± 21.8 nm, respectively) was observed.

### The TSP8 domain is essential for ADAMTS13 binding to the D4-CK domains of VWF and conformational activation

Using the same equilibrium plate binding assay, minimal binding was observed between VWF D4-CK and either WT MDTCS or WTΔTSP8-CUB2 (Fig. 4*E*), indicating TSP8 as a major VWF D4-CK binding partner. Indeed, the in-frame deletion of TSP8 (WTΔTSP8) also largely reduced binding (*K_D_*_(APP)_ of ∼1 μm).

**Figure 5.**
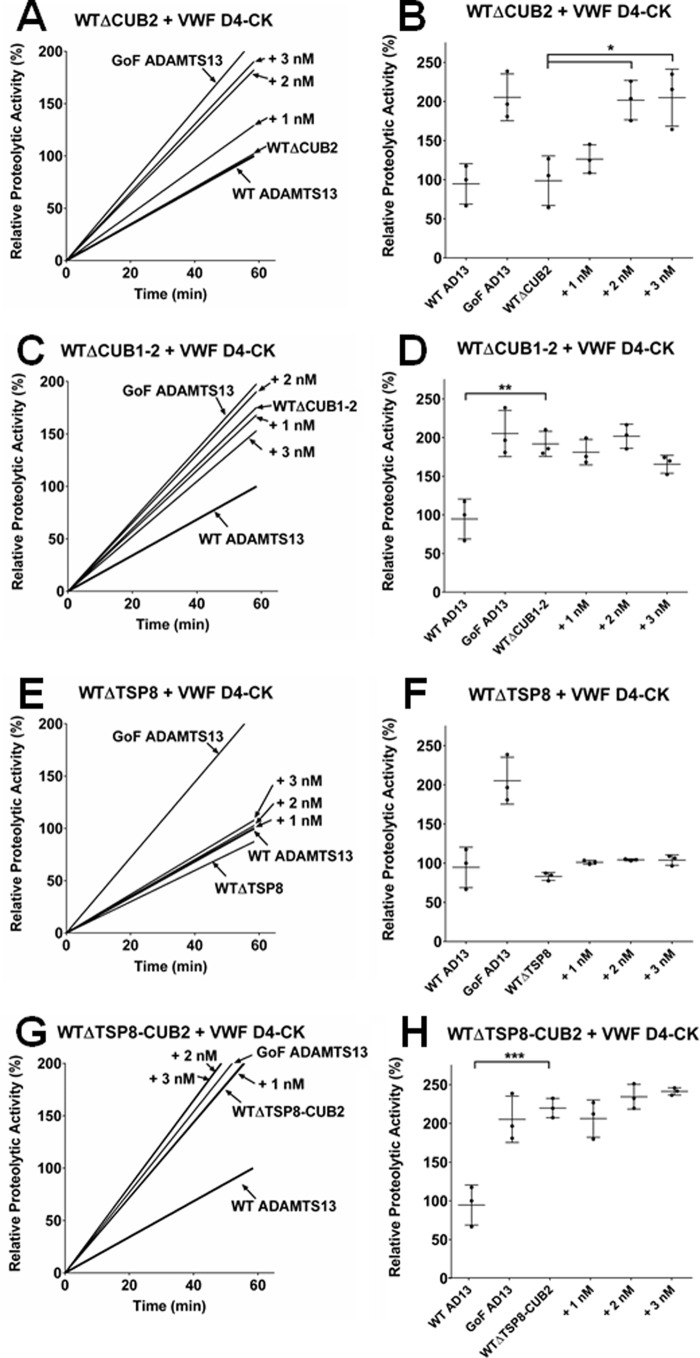
**TSP8 was not required to maintain the closed ADAMTS13 conformation but was essential for conformational activation by VWF D4-CK.** The FRETS-VWF73 assay was used to determine the relative proteolytic activities of the C-terminal truncated ADAMTS13 variants WTΔCUB2 (*A* and *B*), WTΔCUB1-2 (*C* and *D*), WTΔTSP8 (*E* and *F*), and WTΔTSP8-CUB2 (*G* and *H*). The ability of VWF D4-CK (added at 1, 2, or 3 nm) to enhance the activities of these variants was also examined. In each panel the activities of WT and GoF ADAMTS13 are included for reference. Normalized raw data (*A*, *C*, *E*, and *G*) is representative of three independent experiments and relative proteolytic activities (*B*, *D*, *F*, and *H*) are presented as the mean ± S.D. (*n* = 3). *, *p* < 0.05; **, *p* < 0.01; ***, *p* < 0.005.

We studied next the importance of the three ADAMTS13 domains established as able to bind to VWF D4-CK (CUB1, CUB2, and TSP8) for conformational activation using the FRETS-VWF73 assay. As in earlier experiments (Fig. 2*D*), removal of the CUB2 domain did not induce hyperactivity compared with WT ADAMTS13 (Fig. 5, *A* and *B*). Moreover, WTΔCUB2 activity was significantly enhanced (*p* < 0.05) in a dose-dependent manner by the addition of VWF D4-CK, similar to the enhancement observed previously for WT ADAMTS13 (31).

The increased activity of truncated ADAMTS13, lacking both CUB1 and CUB2, is now established (Fig. 2*D*) and previously (31). This is reflected in the results presented in Fig. 5, *C*, *D*, *G*, and *H*, in which both WTΔCUB1-2 and WTΔTSP8-CUB2 exhibited a level of activity similar to that of GoF ADAMTS13. Also, neither of these variants was further activated by the addition of VWF D4-CK. Interestingly, the in-frame deletion of TSP8 did not exhibit enhanced activity. However, its activity was also not enhanced by VWF D4-CK (Fig. 5, *E* and *F*).

## Discussion

It had long been assumed that ADAMTS13 is secreted into the plasma in a constitutively active state and that its proteolytic activity against VWF is regulated solely by its dependence on the shear-induced conformational change in the VWF A2 domain. Recent work has indicated that ADAMTS13 itself undergoes a conformational change required for it to achieve full activity (31, 32). Using electron microscopy (31) and small angle X-ray scattering (32), it was shown that ADAMTS13 can adopt a folded conformation with its C-terminal domains in close proximity to its N-terminal MDTCS domains. Using a GoF ADAMTS13 variant (34), which is constitutively active, and truncated ADAMTS13 variants (WT and GoF MDTCS), it was demonstrated that autoinhibition of ADAMTS13 arises from binding between the CUB1-2 domain fragment and the Arg-568, Phe-592, Arg-660, Tyr-661, and Tyr-665 residues of its spacer domain (31).

To further delineate the autoinhibitory interaction between the spacer domain and C-terminal domains of ADAMTS13, we have used a combination of SPR and co-IP to study binding of the isolated CUB1 and CUB2 domain fragments to MDTCS. Surprisingly, considering the minimal surface area of the five spacer domain residues (Arg-568/Phe-592/Arg-660/Tyr-661/Tyr-665), both CUB1 and CUB2 exhibited independent binding to WT MDTCS (Fig. 1, *A* and *B*). The affinities of CUB1 and CUB2 binding to MDTCS (135 ± 10.1 nm and 86.9 ± 9.0 nm, respectively) were lower than that of the CUB1-2 domain fragment reported here (Fig. 1, *C* and *G*) and previously (31). The 2-fold increase in the B_max_ of CUB1-2, compared with the isolated CUB1 and CUB2 domains, suggests that when immobilized the MDTCS binding sites on CUB1 and CUB2 can both be occupied by MDTCS. As expected, the binding between CUB1 and MDTCS was completely abolished in the GoF MDTCS variant (Fig. 1*D*). However, minimal binding between CUB2 and GoF MDTCS was observed (Fig. 1*E*).

**Figure 6.**
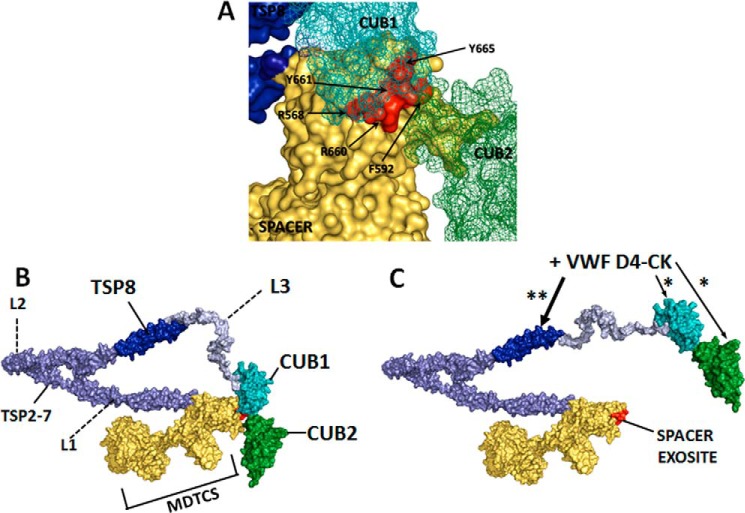
**The proposed mechanism of ADAMTS13 conformational activation.** *A*, the CUB-spacer domain interface within a three-dimensional model of ADAMTS13. The spacer domain exosite 3 residues, mutated in the GoF ADAMTS13 variant, are indicated and shown in *red. B*, the closed conformation of ADAMTS13 showing the relative positions of the N-terminal domains MDTCS and the C-terminal CUB domains. Folding of the TSP2–8 domains is facilitated by the three flexible linker regions between TSP2 and TSP3 (*L1*), between TSP4 and TSP5 (*L2*), and between TSP8 and CUB1 (*L3*). *C*, moderate affinity interactions (*) between the D4-CK domains of VWF and CUB1/CUB2 serve to orientate VWF. As the binding interaction (**) occurs between TSP8 and the D4-CK domains of VWF, the rigid structure of the D4-CK domains causes a repositioning of the CUB domains at the L3 linker region. In the open conformation of ADAMTS13 the spacer domain exosite is exposed, in preparation for VWF A2 domain unfolding. The three-dimensional model of ADAMTS13 was constructed using the DTCS crystal structure (PDB 3GHM; Ref. 39), homology modeling of the MP-Dis domains, homology modeling of the CUB domains, and the thrombospondin-1 (TSP) type 1 repeat crystal structure (PDB 1LSL; Ref. 40).

Having established that both CUB1 and CUB2 interact with the spacer domain exosites, we next examined the autoinhibitory potential of these isolated CUB domains using FRETS-VWF73 assays. As reported previously (31), both WT and GoF MDTCS exhibited an ∼2-fold increase in activity compared with WT ADAMTS13, similar to that of GoF ADAMTS13 (Fig. 2, *A–C*). Despite the evidence that both CUB domains bind to the spacer domain exosite, only CUB1 appeared to exert an inhibitory effect, with both the CUB1 and CUB1-2 domain fragments inhibiting WT MDTCS activity to a similar extent (Fig. 2*C*). Neither CUB1 nor CUB1-2 was able to inhibit GoF MDTCS, as would be expected from the binding studies, and any residual binding that may exist between CUB 2 and GoF MDTCS was not inhibitory. The autoinhibitory potential of CUB1 was corroborated by determining the activities of C-terminal-truncated ADAMTS13 variants (Fig. 2*D*), as increased activity was only achieved in the WTΔCUB1-2 variant. The predicted relative geometry of the CUB domains and MDTCS (Fig. 6*A*) would allow for binding between the spacer domain exosites and both CUB1 and CUB2. When originally described by Jian *et al.* (34), it appeared to be the Y661F and Y665F substitutions (in the variants M4 and M5) that predominantly resulted in hyperactivity of ADAMTS13. In our proposed model, CUB1 is in close proximity to Tyr-661 and Tyr-665 (Fig. 6*A*). It is, therefore, likely that binding between CUB1 and the Tyr-661/Tyr-665 residues (which is abolished in their M4 and M5 variants) is responsible for maintaining the closed ADAMTS13 conformation.

In previous studies of ADAMTS13 conformational activation (31, 32) it was shown that the conformational change, required for ADAMTS13 to achieve full activity, is induced by binding to its substrate, particularly the D4-CK domains of VWF. Exactly how VWF binding brings about this conformational change is not known. A simple explanation is that the D4-CK domains bind to the CUB domains of ADAMTS13 (particularly the autoinhibitory CUB1 domain), competing for the spacer domain binding site. Using SPR we have now established that both CUB1 and CUB2 are able to bind to the VWF D4-CK domain fragment (Fig. 3, *A–D*). However, the affinity of binding between CUB2 and VWF D4-CK is lower than that of CUB1. This may be the result of CUB2 being coupled to the chip in a suboptimal orientation, a suggestion supported by the reduction in its B_max_. This moderate affinity interaction between CUB1-2 and D4-CK (*K_D_* of ∼50 nm) is similar to that previously established for the interaction between CUB1-2 and WT MDTCS (31) and could be sufficient to overcome the closed conformation.

However, SPR analysis of the C-terminal-truncated ADAMTS13 variants WTΔCUB2 and WTΔCUB1-2 (Fig. 4, *A–D*) has shown that removal of the CUB domains resulted in a significant reduction in affinity compared with WT ADAMTS13 (*p* < 0.05) but it did not completely eliminate binding to VWF D4-CK. A similar magnitude (but statistically insignificant) decrease in affinity for VWF D4-CK was also observed in equilibrium plate binding assays, suggesting binding can occur in the absence of CUB1 and CUB2 (Fig. 4*E*). In this assay, *K_D_*_(APP)_ values could not be determined for either WT MDTCS (lacking the entire C terminus) or WTΔTSP8-CUB2 (truncated after TSP7) due to their weak binding. This strongly suggested TSP8 as a major binding site for VWF D4-CK. Indeed, the in-frame domain deletion of TSP8 (WTΔTSP8) produced a strong decrease in *K_D_*_(APP)_ (*p* < 0.001). The importance of both TSP8 and the CUB domains in ADAMTS13 activation by VWF D4-CK was also evident in FRETS-VWF73 assays (Fig. 5, *A–H*). The variant WTΔCUB2, with activity similar to WT ADAMTS13, was enhanced by the addition of VWF D4-CK in a dose-dependent manner similar to that seen previously for WT ADAMTS13 (31), confirming that the interaction between CUB2 and VWF D4-CK is not required for ADAMTS13 activation. As expected, the variants WTΔCUB1-2 and WTΔTSP8-CUB2, both lacking the CUB1 domain, had enhanced activity and could not be further enhanced by the addition of VWF D4-CK. Interestingly, the in-frame deletion of TSP8 did not appear to increase its activity (Fig. 5*F*). However, no enhancement of activity was observed upon the addition of VWF D4-CK, indicating that the interaction between TSP8 and VWF D4-CK is a crucial component of the activation mechanism.

On the basis of the results presented herein we propose a model of the molecular mechanism of ADAMTS13 conformational activation involving multiple interactions between the D4-CK domains of VWF and the C-terminal domains of ADAMTS13 (Fig. 6). In the closed ADAMTS13 conformation (Fig. 6*B*) both CUB1 and CUB2 interacted with the spacer domain exosite, with the autoinhibitory CUB1 domain potentially positioned to interact with the key residues Tyr-661 and Tyr-665 (Fig. 6*A*). The positioning of the CUB domains is facilitated by a folded conformation of the TSP2-TS8 domains around the three flexible linker regions (33). A moderate affinity interaction occurs between the CUB domains and one or more of the VWF D4-CK domains, suggested previously to be the VWF D4 domain (17) (Fig. 6*C*). The affinity of this interaction may be sufficient to compete for binding of the CUB domains to the spacer domain, potentially reducing the stability of the closed conformation. It may also serve to position the D4-CK domains to facilitate the higher affinity interaction between VWF and TSP8, shown here to be critical for activation. The dimeric D4-CK domains of VWF have been shown by EM to adopt a rigid stem-like conformation maintained by specific pair-wise interactions between the densely packed domains (38). As VWF D4-CK binds to TSP8 while simultaneously binding to the CUB domains to weaken the CUB-spacer interaction, the rigidity of the D4-CK domains would be expected to cause a change in position of the CUB domains facilitated by the flexible L3 linker region, bringing them parallel to the TSP5–8 domains (Fig. 6*C*). Ultimately, this conformational reorientation would fully expose the spacer domain exosite in readiness for VWF A2 domain unfolding.

Previously, it has been shown that conformational activation of ADAMTS13 is an important consideration in understanding the pathophysiological mechanism of autoantibody development in TTP (31). More recently it has also been suggested that conformational quiescence of ADAMTS13 serves to protect other plasma proteins from off-target proteolysis in circulation (35). Further investigation into the mechanism of conformational activation may provide new insights into these physiological processes and may be an important consideration as ADAMTS13, particularly conformationally activated variants, are developed as potential therapeutic agents for the treatment of ischemic stroke and other cardiovascular diseases.

## Experimental procedures

### Production of recombinant ADAMTS13 fragments

Recombinant human ADAMTS13 with a C-terminal Myc/His_6_ tag in pCDNA3.1 (36) was used to generate GoF ADAMTS13 (R568K/F592Y/R660K/Y661F/Y665F) by sequential site-directed mutagenesis. The same mutagenesis primers were also used to introduce the GoF mutations into WT MDTCS (ADAMTS13 residues 1–685) with a C-terminal Myc/His_6_ tag in pCEP4. The truncated variants WTΔCUB1-2 (ADAMTS13 residues 1–1191), WTΔCUB2 (ADAMTS13 residues 1–1298), WTΔTSP8-CUB2 (ADAMTS13 residues 1–1075), GoFΔCUB1-2 and GoFΔCUB2, the in-frame deletion WTΔTSP8 (ADAMTS13 residues 1–1075, 1131–1427), and the CUB1-2 (ADAMTS13 residues 1192–1427), CUB1 (ADAMTS13 residues 1192–1298), and CUB2 (ADAMTS13 residues 1299–1427) domain fragments were generated by inverse PCR using the full-length ADAMTS13 constructs. All ADAMTS13 variants and fragments were expressed in HEK293S cells, purified, and quantified using in-house ELISAs as described previously (36, 37). The dimeric VWF D4-CK domain fragment (VWF residues 1874–2813) in the vector pcDNA 3.1/His was transiently expressed in HEK293S, purified by FPLC, and quantified by ELISA as previously described (17).

### Surface plasmon resonance and equilibrium plate binding assays

Binding between ADAMTS13 (and its variants), the isolated CUB domains and the VWF D4-CK domain fragment was determined by surface plasmon resonance using a BIAcore T100 system as previously described (17). All SPR experiments were performed in 10 mm HEPES, 150 mm NaCl at pH 7.4 in the presence of 0.0034 mm EDTA, 0.005% Tween 20 to reduce nonspecific binding between the analyte and the dextran matrix on the CM5 chip. Binding between ADAMTS13 (and its variants) and VWF D4-CK was also determined by equilibrium plate binding in PBS, 0.1% BSA at pH 7.4 as described (17, 24). Briefly, 50 nm ADAMTS13 (and its variants) in 50 mm sodium carbonate buffer (pH 9.6) were immobilized onto the wells of a 96-well microtiter plate (Nunc). Increasing concentrations of purified VWF D4-CK (0–500 nm) were applied for 2 h at 37 °C. Bound VWF D4-CK was detected using a HRP-conjugated α-VWF pAb (Dako). Binding curves were fitted to the one-binding site model using GraphPad Prism 7 software to determine *K_D_*_(APP)_.

### Purification of domain-specific α-ADAMTS13 antibodies

Domain-specific antibodies were affinity-purified from a preparation of total IgG purified from rabbits immunized with full-length recombinant WT ADAMTS13. Briefly, recombinant MP-Dis and CUB1 fragments were immobilized on activated NHS Hitrap columns (GE Healthcare) and used to deplete the IgG pool of antibodies against these domains. Once depleted of α-CUB1 antibodies, the same IgG pool was passed over an immobilized recombinant CUB1-2 fragment to purify antibodies specific to CUB2. The antibody preparations were assessed for purity by SDS-PAGE and quantified by Nanodrop. The specificity of each preparation was confirmed by SDS-PAGE and Western blot of ADAMTS13 fragments using the prepared antibodies (1:1000 dilution) followed by HRP-conjugated goat α-rabbit secondary antibody (Sigma).

### Co-IP analysis of CUB-MDTCS interactions

WT or GoF MDTCS (40 nm) in the absence/presence of 40 nm CUB1, CUB2, or CUB1-2 fragment were prepared in PBS, 0.1% BSA, and 0.02% Tween 20 at pH 7.4 and immunoprecipitated using Protein G Dynabeads (Invitrogen) coupled to antibodies against either the MP-Dis, CUB1, or CUB2 domains of ADAMTS13. Beads were washed, and bound protein was eluted in 1× LDS loading buffer at 70 °C for 10 min. Inputs (pre-IP), flow-through (post-IP), and bound protein in eluates were analyzed by SDS-PAGE and Western blot using a HRP-conjugated anti-His_6_ antibody (Abcam). To exclude any confounding effect of pH on the CUB-MDTCS interaction, a co-IP analysis of binding between MDTCS and CUB1-2 was performed at both pH 7.4 and pH 6; essentially identical results were obtained.

### ADAMTS13 activity assays

For FRETS-VWF73 assays ADAMTS13, MDTCS, ΔCUB2 or ΔCUB1-2 variants (WT and GoF), and WTΔTSP8 or WTΔTSP8-CUB2 were diluted to 0.5 nm in reaction buffer (5 mm Bis-Tris, pH 6.0, 25 mm CaCl_2_, 0.005% Tween 20) in white 96-well plates (Nunc). Purified CUB1, CUB2, or CUB1-2 domain fragments (6 nm) or VWF D4-CK (2–6 nm) was added before a 45-min preincubation at 37 °C. The reaction was initiated by the addition of an equal volume of 4 μm FRETS-VWF73 substrate (Peptanova GMBH). Fluorescence (excitation 340 nm; emission 460 nm) was measured at 37 °C at 1-min intervals for 1 h using a Fluostar Omega plate reader (BMG Labtech). Results were normalized to WT ADAMTS13 activity. To exclude any confounding effects of pH on the activation of ADAMTS13 by VWF D4-CK, the relative activities of WT ADAMTS13 (in the absence/presence of 6 nm VWF D4-CK) and GoF ADAMTS13 were determined at pH 6, 6.5, 7, and 7.5. Enhanced activity of WT ADAMTS13 induced by VWF D4-CK was observed at each pH.

## Author contributions

K. S. and M. O. F. performed the research. K. S. and D. A. L designed the research, analyzed the data, interpreted the data, generated the figures, and wrote the paper.

## Footnotes

3 The abbreviations used are:

von VWF: Willebrand factor
TTP: thrombotic thrombocytopenic purpura
GoF: gain of function
SPR: surface plasmon resonance
RU: response units
co-IP: co-immunoprecipitation
Bis-Tris: 2-[bis(2-hydroxyethyl)amino]-2-(hydroxymethyl)propane-1,3-diol.
